# *Ixodes* Immune Responses Against Lyme Disease Pathogens

**DOI:** 10.3389/fcimb.2018.00176

**Published:** 2018-05-29

**Authors:** Chrysoula Kitsou, Utpal Pal

**Affiliations:** Department of Veterinary Medicine and Virginia-Maryland Regional College of Veterinary Medicine, University of Maryland, College Park, MD, United States

**Keywords:** *Ixodes* ticks, immunity, *Borrelia burgdorferi*, lyme disease, microbial recognition

## Abstract

Although *Ixodes scapularis* and other related tick species are considered prolific vectors for a number of important human diseases, many aspects of their biology, microbial interactions, and immunity are largely unknown; in particular, how these ancient vectors recognize invading pathogens like *Borrelia burgdorferi* and influence their persistence. The analysis of the *Ixodes* genome and a limited set of transcriptomic data have established that ticks encode many components of classical immune pathways; yet at the same time, they lack many key orthologs of these recognition networks. Therefore, whether a given immune pathway is active in *Ixodes* ticks and how precisely they exert its microbicidal functions are only incompletely delineated. A few recent studies have suggested that classical pathways like the Janus Kinase/Signal Transducer and Activator of Transcription (JAK/STAT) as well as immunodeficiency (IMD) pathways are fully functional in *I. scapularis*, and upon challenge with microbes, generate potent microbicidal responses against diverse tick-borne pathogens including *B. burgdorferi*. These studies also highlight novel concepts of vector immunity that include both a direct and an indirect mode of recognition of pathogens, as well as the influence of the gut microbiome, which ultimately dictates the outcome of a robust microbicidal response. Further understanding of how *Ixodes* ticks recognize and suppress invading microbes like *B. burgdorferi* will enrich our fundamental knowledge of vector immunobiology, thereby contributing to the development of future interventions to better control the tick-borne pathogen.

## Introduction

Ticks are ancient, yet highly adapted hematophagous organisms that have exquisitely evolved to parasitize a wide array of vertebrate hosts, including extinct reptiles like dinosaurs (Peñalver et al., 2017). Therefore, it is not surprising that ticks transmit several distinct pathogens to humans or domesticated animals than any other arthropod vector (Jongejan and Uilenberg, 2004; Paddock et al., 2016). Ticks were first documented as a parasite to humans by ancient Greeks (Sonenshine, 1993; Parola and Raoult, 2001), while in the modern era, their role as disease vectors was recognized in the late eighteenth century (Smith and Kilborne, 1893). While there are many species of ticks known to transmit diseases to human and animals, *Ixodes* ticks are one of the most efficient disease vectors, aided by a widespread distribution across the globe (Piesman and Gern, 2004). Out of the 15 tick-borne infections reported in the U.S., at least six are transmitted by the *Ixodes* tick. Lyme disease and rickettsial diseases are the most predominant *Ixodes*-borne infections that are rapidly emerging as not only the most frequent causes of preventable diseases in the U.S. but also globally (Parola and Raoult, 2001; Brett et al., 2014; Hook et al., 2015). An estimate from the CDC suggests that possibly 300,000 new cases of Lyme disease occur in the U.S. per year (Mead, 2015). In Europe, there are 85,000 cases reported every year although these numbers are likely to be severely underestimated (Cook, 2015). While human vaccines to prevent tick-borne infections, including Lyme disease, are mostly unavailable, their development would require a thorough and mechanistic understanding of the persistence of tick-borne pathogens through its enzootic transmission cycle. The events of how a tick vector recognizes and mounts microbicidal responses are critical for understanding the complex biology of tick-borne infections, tick-pathogen interactions, and the development of new and effective control measures.

The arthropod immune system displays a remarkable diversification across species (Palmer and Jiggins, 2015). They are however, capable of recognizing microorganisms by germ line-encoded, non-rearranging receptors, controlling infection through the generation of rapid microbicidal effector responses (Hoffmann et al., 1999; Hoffmann, 2003; Pham et al., 2007; Myllymäki et al., 2014). Studies over the past several decades have enriched our fundamental knowledge of arthropod immunity, mostly involving model insects like fruit flies (Hoffmann et al., 1999; Irving et al., 2001; Hoffmann and Reichhart, 2002; Hetru et al., 2003; Hoffmann, 2003; Bischoff et al., 2006; Ferrandon et al., 2007; Lemaitre and Hoffmann, 2007) and mosquitoes (Cohuet et al., 2006; Clayton et al., 2014; Smith et al., 2014; Molina-Cruz et al., 2016). Like other arthropods, many disease vectors recognize invading microorganisms using a sophisticated array of immune surveillance mechanisms that allows them to mount an effective immune response. These microbicidal responses are often orchestrated through classical immune signaling pathways like immunodeficiency (IMD), Janus Kinase/Signal Transducer and Activator of Transcription (JAK/STAT) and Toll signaling pathways (Hoffmann et al., 1999; Irving et al., 2001; Hoffmann and Reichhart, 2002; Hoffmann, 2003; Myllymäki et al., 2014). Despite these advances in model insects, our knowledge of vector immunobiology in non-model arthropods, including *Ixodes* ticks is still limited (Smith and Pal, 2014; Oliva Chávez et al., 2017). As comparative genomics studies reveal the existence of remarkable diversity in the immune system of arthropods, it is likely that the tick immune system, of ancient origin, is unique and likely to differ from classical model insects. In addition, as ticks are phylogenetically distant (Sonenshine, 1993; Gulia-Nuss et al., 2016) from dipteran arthropods, such as *Drosophila* and mosquitoes, and the components of the immune system can diversify very rapidly due to pathogen-specific adaptation (Mayer et al., 2016), experimental studies are warranted to reveal tick immune responses against the pathogens they harbor and transmit. In fact, a few recent studies (De Silva, 2016; Smith et al., 2016; Shaw et al., 2017) have discovered the existence of atypical immune signaling components and cascades in ticks. In this review, we will highlight the specificities of the immune response of *Ixodes* ticks to invading microorganisms, including those raised against bacterial pathogens like *B. burgdorferi*.

## Immune signaling pathways encoded in the *Ixodes* genome

The recently described genome of the *I. scapularis* tick reveals the presence of 20,486 mRNA-coding genes, including many gene-products known to involved in vector-host interactions and immunity (Gulia-Nuss et al., 2016). A comprehensive analysis of the *Ixodes* genome suggested the occurrence of a remarkable diversity within classical immune-related genes and related pathways. Most components of the Toll, IMD, JAK-STAT immune signaling cascades and the RNA interference or antiviral pathways were identified in the *Ixodes* genome (Gulia-Nuss et al., 2016). In a previous study, a total of 234 *Ixodes* genes were categorized into nine major immune pathways that included “gut-microbe homeostasis, agglutination, leucine-rich repeat proteins, proteases, coagulation, non-self recognition and signal transduction, free radical defense, phagocytosis, and antimicrobial peptides” (Smith and Pal, 2014). The hemocyte, which is a major “immune” cell in arthropods (Browne et al., 2013), expressed many transcripts associated with known functions, including signal transduction associated with immune responses and transcription factors, such as STAT. In a separate study, Kotsyfakis et al. characterized the transcriptome of hemocytes from female *I. ricinus* where 327 genes were found to be highly expressed, including genes encoding for “scavenger receptors, antimicrobial peptides, pathogen recognition proteins, proteases and protease inhibitors” (Kotsyfakis et al., 2015).

## An indirect, cross-species interferon-like signaling pathway

In a recent investigation of *B. burgdorferi* acquisition in vector ticks, Smith *et al* discovered a novel cross-species signaling cascade that enables *I. scapularis* ticks to sense a mammalian cytokine present in the ingested blood meal as a “signal” for infection (Smith et al., 2016). The signal, via recognition of mammalian interferon by unidentified tick receptor(s), activates a microbicidal response to limit the proliferation of invading *B. burgdorferi* (De Silva, 2016; Smith et al., 2016). The study demonstrated that IFNγ present in the blood meal induces the tick STAT protein leading to the upregulation of an *Ixodes* GTPase (termed as IGTPase) as well as downstream borreliacidal responses by activation of a domesticated amidase effector called Dae2 (Chou et al., 2015) that ultimately affects *B. burgdorferi* persistence in ticks (Smith et al., 2016). Together, these studies highlight the importance of an “indirect” or “host-centric” recognition of infection via sensing of mammalian cytokines in engorged ticks.

## An atypical IMD pathway

The recent availability of tick genome sequencing data and comparative analysis established that ticks lack many key orthologs of major innate immunity cascades like the IMD pathway (Gulia-Nuss et al., 2016; Smith et al., 2016; Oliva Chávez et al., 2017; Shaw et al., 2017). In addition, some of the orthologs in classical immune pathways also display substantial sequence variation that may be reflective of pathogen-specific diversification (Gulia-Nuss et al., 2016; Oliva Chávez et al., 2017; Shaw et al., 2017). However, despite showing a significant divergence and lacking orthologs of many key elements, including the gene encoding an IMD molecule, the pathway remains fully functional and, upon challenge with microbes, generates potent microbicidal responses against diverse tick-borne pathogens like *B. burgdorferi* or *Anaplasma phagocytophilum* (Shaw et al., 2017). Even in the absence of classical cell signaling receptors, including peptidoglycan recognition proteins and the adaptor molecules Fas-associated protein with a death domain and IMD, the pathway is nevertheless, active. The protein-protein interactions between an apoptosis protein, an E3 ubiquitin ligase and E2 conjugating enzyme are critical for the activation of an atypical IMD network in ticks (Shaw et al., 2017). Using RNAi-mediated know-down of several tick gene targets, such as bendless/uev1a, relish, and caspar, the authors show that the IMD pathway reduce colonization of both *B. burgdorferi* and *A. phagocytophilum* in ticks (Shaw et al., 2017). The study identified that infection-derived Diaminopimelic acid (DAP)-type peptidoglycans present in most Gram-negative bacteria as well as specific microbial lipids like “1-palmitoyl-2-oleoyl-sn-glycero-3-phosphoglycerol (POPG) and 1-palmitoyl-2-oleoyl diacylglycerol (PODAG)” that present in *A. phagocytophilum* can stimulate the atypical IMD pathway in ticks (Shaw et al., 2017). Whether similar infection-derived lipids also trigger the IMD pathway during *B. burgdorferi* infection in *I. scapularis* or how specific tick immune responses differs during infection with distinct Gram-negative bacterial agents remain unknown.

## Other pathways: toll and JAK-STAT

The Toll signaling pathway in arthropods is activated in the presence of bacterial, viral, and fungal pathogens (Valanne et al., 2011). Activation of these pathways in arthropods leads to the processing of an extracellular protein, Spätzle, which subsequently activate the signal transduction events leading to the induction of the NF-κ B transcription factor family members Dif and Dorsal—the immune components analogous to mammalian c-Rel and RelA; these events ultimately results in the upregulation of genes encoding specific antimicrobial peptides (AMPs) (Tzou et al., 2002; Tanji et al., 2007; Valanne et al., 2011). Except for a few, most components of insect Toll signaling cascade are encoded in the tick genome. In fact, there are at least 33 genes exist in the *Ixodes* genome that annotated for this pathway (Smith and Pal, 2014; Oliva Chávez et al., 2017), including a gene for MyD88 (ISCW008802) and a few Tolls (such as ISCW007724 and ISCW017724) that are dramatically induced during *B. burgdorferi* infection in *Ixodes* ticks (Smith et al., 2016). While evidence for functional Toll signaling exists in non-*Ixodes* ticks like *Rhipicephalus microplus* (Rosa et al., 2016), whether and how the Toll pathway operates in *Ixodes* ticks remain largely obscure. In *Drosophila*, upregulation of Toll pathway genes leads to the generation of specific AMPs (Imler and Bulet, 2005; Tanji et al., 2007; Shandala and Brooks, 2012), such as drosomycin, defensing, and other AMPs (Sonenshine et al., 2002; Hynes et al., 2005). Ticks also encode several defensin-like AMPs (Smith and Pal, 2014) but whether these are regulated by Tolls or there is other Toll-specific AMP that controls bacterial pathogens like *B. burgdorferi* is unknown.

The Janus Kinase/Signal Transducer and Activator of Transcription pathway (JAK/STAT) also remains less well-studied in ticks, although comparative genomics studies have revealed that the pathway is conserved between ticks and fruit flies (Agaisse and Perrimon, 2004; Rawlings et al., 2004; Smith and Pal, 2014; Gulia-Nuss et al., 2016). While key components of the classical JAK/STAT pathway are absent in *Ixodes* ticks, as further discussed below, they still actively utilize this pathway to suppress invading pathogens like *A. phagocytophilum* (Liu et al., 2012). Independent RNAi-mediated knockdown of STAT or a downstream 5.3-kDa antimicrobial peptide affects the persistence of *A. phagocytophilum* in ticks (Liu et al., 2012). The JAK/STAT pathway, which is essential for recognition and response to bacteria in fruit flies, is activated by the transmembrane receptor Dome via binding of a cytokine-like signaling molecule, Unpaired (Upd) (Ghiglione et al., 2002; Agaisse and Perrimon, 2004; Tsai and Sun, 2004; Hombría et al., 2005). While the *Ixodes* genome encodes for at least four Dome orthologs, an ortholog gene encoding Upd, which triggers classical JAK/STAT pathway in insects, is noticeably absent in ticks (Smith and Pal, 2014; Gulia-Nuss et al., 2016; Oliva Chávez et al., 2017). Therefore, given the absence of a canonical Dome ligand like Upd, *Ixodes* likely uses other atypical ligand(s) to active the pathway, which remain to be identified.

## Microbiome and immune responses

The roles of gut microbiota in the physiology and immune homeostasis in diverse organisms are now well-established (Ley et al., 2008; Round and Mazmanian, 2009; Hooper et al., 2012; Buchon et al., 2013; Kamada et al., 2013). The tick microbiome has been characterized in many studies, and shown to be comprised of both intra and extracellular bacteria representing up to108 distinct genera, including several symbionts like *Coxiella, Midichloria, Francisella, Wolbachia, Cardinium, Arsenophonus, Spiroplasm*a, *Rickettsi*a, *Rickettsiella*, and *Lariskella* (Andreotti et al., 2011; Carpi et al., 2011; Menchaca et al., 2013; Bonnet et al., 2017; Swei and Kwan, 2017). Notably, the abundance of a specific genus represented in tick microbiota could alter during infection with a specific pathogen; for example, while the relative occurrence of *Rickettsia* and *Enterococcus* were decreased during *A. phagocytophilum* colonization of *I. scapularis* ticks, on the other hand, the level of *Pseudomonas* was enhanced (Abraham et al., 2017; Narasimhan et al., 2017). A series of new studies have begun to unravel the role of tick gut microbiota on the persistence of tick-borne pathogens, including *B. burgdorferi*. Recent reports have also highlighted the impact of the host blood meal on the diversity of the microbiome in *Ixodes* ticks that influences vector competency and ultimately impacts pathogen persistence (Abraham et al., 2017; Narasimhan et al., 2017). As highlight in these studies, infection-induced alterations in the tick gut microbiota could impair the tick structural barriers like integrity of the peritrophic matrix and possibly tick immune signaling pathway, ultimately allowing tick-borne pathogens to more effectively colonize the vector (Abraham et al., 2017). It is however, currently unknown precisely how the gut microbiota influences tick immune responses, including cross-species host blood meal-derived cytokine signaling events, that are likely to impact the ability of *Ixodes* ticks to recognize and control microbial infection.

## Immune barrier—the dityrosine network

A gene encoding a nicotinamide adenine dinucleotide phosphate oxidase, termed dual oxidase (Duox), has been shown to support host-microbe homeostasis (Ha et al., 2005, 2009). In the *Drosophila* gut, Duox generates reactive oxygen species (ROS) in a way that fine tunes microbial burdens, thereby controlling the over-proliferation of gut bacteria (Ha et al., 2009). However, in spite of generating ROS production, Duox also catalyzes the cross-linking of the extracellular matrix (ECM) molecules via tyrosine residues (Edens et al., 2001). This, in turn, aids to stabilize the ECM and assists in the formation of an acellular gut barrier, known as the dityrosine network (DTN). The molecular lining is distinct yet analogous to another well-known and relatively more rigid gut barrier called peritrophic matrix (PM) described in most insects including ticks (Zhu et al., 1991; Kariu et al., 2013). The PM is induced in *Ixodes* ticks during blood feeding and forms a mechanical barrier between gut lumen and adjacent epithelial cells. While the PM is likely to be more associated with digestive physiology of the gut, the DTN, on the other hand, is involved in maintenance of immune homeostasis in gut, via involvement of the Duox. In *Drosophia*, this enzyme functions as an integral part of the fly protective immunity since in its absence, the flies became more susceptible bacterial toxins (Ha et al., 2005). In the *Anopheles gambiae* gut Duox, together with a gut peroxidase, catalyzes the formation of the DTN, which surrounds the gut epithelial layer (Kumar et al., 2010). Replication of commensal gut bacteria during blood meal engorgement could activate epithelial immunity. However, genesis of DTN reduces the gut permeability to immune elicitors by shielding the gut microbiota that ultimately benefit the invading pathogens (Kumar et al., 2010). The annotated *I. scapularis* genome harbors one Duox and 16 peroxidase genes (Smith and Pal, 2014), out of which at least 11 peroxidases are expressed during tick engorgement process (Yang et al., 2014). In a recent study Duox, along with a specific gut peroxidase (ISCW017368), was shown to be involved in DTN formation in ticks (Yang et al., 2014). This barrier benefit microbial survival in the gut, as reduced DTN as resulting from the silencing of Duox or the specific peroxidase impairs *B. burgdorferi* persistence in ticks. Impaired DTN in RNAi-mediated silenced ticks also activated specific tick innate immune pathways, specifically a nitric-oxide synthase gene-product that potentially results in the reduction of spirochete levels (Yang et al., 2014). Taken together, these observations underscore the biological significance of the DTN in ticks, particularly for its role in protecting invading pathogens like Lyme disease pathogens.

## Concluding remarks

**Figure 1:**
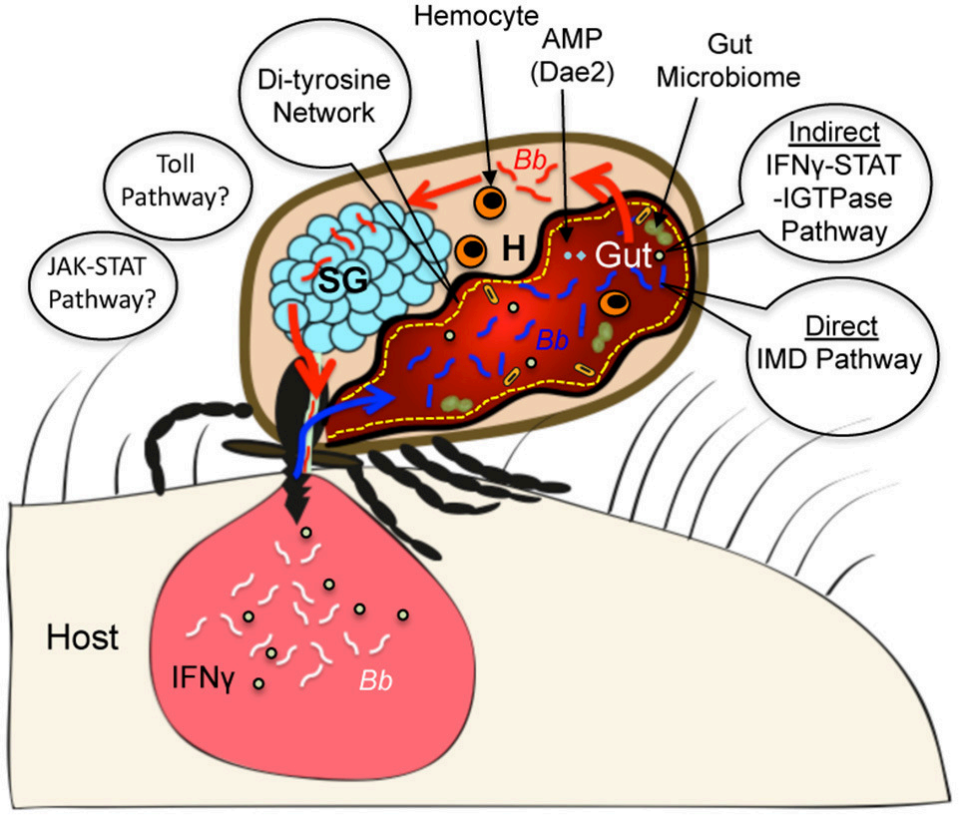
Diagram highlighting representative examples of *Ixodes* immune components that impact persistence of Lyme disease agents. During tick engorgement on a mammalian host, *B. burgdorferi* (*Bb*, white color) from an infected host dermis are acquired in ticks (*Bb* in tick gut indicated in blue color). *Ixodes* ticks can recognize the presence of pathogens in the incoming blood using multiple immune surveillance mechanisms, including an indirect cross-species signaling mechanism that involves sensing mammalian cytokines like IFNγ (indicated by green dots). Ingested cytokine in the tick initiate microbicidal responses involving a tick STAT, an immune GTPase (IGTPase) and an antimicrobial protein (AMP) like Dae2. In addition, a direct recognition mechanism, such as involving an atypical IMD pathway, is also operative in ticks that limits spirochete levels in the vector. The other classical components of innate immune cascades like Toll and JAK-STAT signaling pathways, which are although represented in *Ixodes* genome, their roles in direct recognition of *B. burgdorferi* in *Ixodes* ticks remain unknown. Additional factors in the gut, such as a feeding induced molecular barrier (di-tyrosine network) and the gut microbiome also impact the persistence of spirochetes. During a subsequent blood meal, *Bb* (red color) exit the tick gut and disseminate to salivary gland (SG) via hemocoel (H), which contains hemocytes that can also impact microbial survival in the vector.

Arthropods evolved millions of years earlier than the ancestors of cartilaginous fish where a more sophisticated form of immunity or “adaptive immunity” first appeared (Flajnik and Kasahara, 2010). Thus, these invertebrates, including *Ixodes* ticks, exclusively protect themselves from microbial invasion via innate immune responses. Similar to other arthropods, the tick immune system possibly recognizes microorganisms by innate immune receptors, and suppresses infection via activation of robust antimicrobial responses (Hoffmann and Reichhart, 2002; Hoffmann, 2003; Ferrandon et al., 2007; Valanne et al., 2011; Myllymäki et al., 2014). However, recent studies highlight the existence of atypical microbial recognition mechanisms, including the detection of specific cytokines present in the incoming blood meal from infected hosts, peptidoglycans or specific membrane lipids and mount antimicrobial responses through the participation of specific immune GTPases and microbicidal responses (Smith et al., 2016; Shaw et al., 2017), including involvement of domesticated amidase effectors (Chou et al., 2015). Some of these recent developments in our understanding of tick immune responses against Lyme disease agents are highlighted in a schematic diagram (Figure 1). While more immune mechanisms have been recently shown to exist in flies, including the induction of a systemic antiviral RNAi response (Saleh et al., 2009; Tassetto et al., 2017) or a specific and life-long “memory” immune response against some bacterial species (Agaisse, 2007; Pham et al., 2007), whether pathways are present in ticks remains unknown. In addition, new studies in *Drosophila* and mammals, including humans, also suggest the existence of other alternative immune mechanisms, such as more host-centric or indirect immune pathways for recognition of virulent bacteria by sensing pathogenic-derived effector molecules that modify host target molecules (Boyer et al., 2011; Cui et al., 2015). It is uncertain whether ticks also possess such “effector-triggered” immunity mechanisms able to discriminate between avirulent and virulent microorganisms to defend themselves against microbes with pathogenic potential. Therefore, future studies to understand tick immune responses against invading pathogens are much needed. These studies have the potential to discover novel immune mechanisms but also to provide fundamental knowledge on the evolution over millions of years of the defense mechanisms that have allowed ticks to become highly successful hematophagous parasites and prolific vectors of a number of blood-borne diseases, including Lyme disease.

## Author contributions

All authors listed have made a substantial, direct and intellectual contribution to the work, and approved it for publication.

### Conflict of interest statement

The authors declare that the research was conducted in the absence of any commercial or financial relationships that could be construed as a potential conflict of interest.
